# Cost-effectiveness of sorafenib versus SBRT for unresectable advanced hepatocellular carcinoma

**DOI:** 10.1186/s13014-016-0644-4

**Published:** 2016-05-18

**Authors:** Henry W. C. Leung, Chung-Feng Liu, Agnes L. F. Chan

**Affiliations:** Department of Radiation Therapy, An Nan Hospital, China medical University, No. 66, Sec. 2, Changhe Rd., Annan Dist., Tainan, Taiwan; Department of Nursing, Min-Hwei College of Health Care management, No.111 6, Sec 2, Zongshan E. Rd., Liuying Township, Tainan City, 736 Taiwan; Department of Information Management, Chia Nan University of Pharmacy & Science, No. 60, Sec 1, Erren Rd., Rende Dist., Tainan City, 71710 Taiwan; Department of Pharmacy, An-Nan Hospital, China Medical University, 1. No. 66, Sec. 2, Changhe Rd., Annan Dist., Tainan, Taiwan

**Keywords:** Cost-effectiveness analysis, ICER, Advanced hepatocellular carcinoma, Sorafenib

## Abstract

**Objective:**

Stereotactic body radiotherapy (SBRT) has been shown to improve overall survival in patients with advanced hepatocellular carcinoma. This study aimed to assess the cost-effectiveness of SBRT compared to sorafenib which is the only drug for advanced hepatocellular carcinoma.

**Methods:**

A Markov decision-analytic model was performed to compare the cost-effectiveness of SBRT and sorafenib for unresectable advanced hepatocellular carcinoma. Patients transitioned between three health states: stable disease, progression disease and death. We calculated the data on cost from the perspective of our National Health Insurance Bureau. Sensitivity analyses were conducted to determine the impact of several variables.

**Results:**

The incremental cost effectiveness ratio (ICER) for sorafenib compared to SBRT was NT$3,788,238 per quality-adjusted life year gained (cost/QALY), which was higher than the willingness to pay threshold of Taiwan according to WHO’s guideline. One-way sensitivity analysis revealed that the utility of progression disease for the sorafenib treatment, utility of progression free survival for SBRT, utility of progression free survival for sorafenib, utility of PFS to progression disease for SBRT and transition probability of progression disease to dead for SBRT were the most sensitive parameters in all cost scenarios. The Monte-Carlo simulation demonstrated that the probability of cost-effectiveness at a willingness to pay threshold of NT$ 2,213,145 per QALY was 100 % and 0 % chance for SBRT and sorafenib.

**Conclusion:**

This study indicated that SBRT for advanced hepatocellular carcinoma is cost-effective at a willingness to pay threshold as defined by WHO guideline in Taiwan.

## Background

Hepatocellular carcinoma (HCC) is the second leading cause of cancer death worldwide and Taiwan in 2012 and 2014, respectively [1, 2]. The incidence and mortality of HCC has continuously increased globally in North America and Asian countries [3–5]. The increased incidence of HCC is correlated with the high prevalence of cirrhosis hepatitis C virus, which did not have any vaccine for prevention [6]. HCC can be treated with surgical resection, radiofrequency ablation (RFA), transarterial chemoembolization (TACE) or liver transplantation if diagnosed early [7, 8]. However, the majority of HCC patients are diagnosed at an advanced stage with poor liver function, a significant proportion of patients are incurable, with median survival rate are generally 1-year ranging from 20 to 30 % [9–11]. In patients with un-resectable HCC and contraindicated TACE, sorafenib is the only option that can increase 1-year survival to 45 %, particularly effective in patients with limiting extrahepatic spread [12, 13]. As the advances in technology of radiation planning and imaging, Stereotactic body radiotherapy (SBRT) is a radiation technique which deliver a higher radiation dose in few fractions to target lesions with low doses to the noninvolved liver. Therefore it has become a feasible and safe modern technique for patients with localized HCC and not eligible to TACE [14]. The radiation-induced liver disease (RILD) rates were less than 5 % [15]. Over the past decades, many studies have suggested that SBRT can be used safely with local control rates of 75 to 100 % at 1 to 2 years survival [14, 16–18]. Recently, the phase I and the subsequent phase II trial reported that SBRT improved the median overall survival about 17 months [16].

Although sorafenib and SBRT has showed an improvement in the median overall survival for the treatment of advanced HCC, the financial burden for its use are substantial. As the one payer healthcare system in our country, the healthcare expenditures have become one of the most important issues from the perspective of healthcare provider. This study was intended to compare whether sorafenib or SBRT is cost-effect for patients with inoperable advanced HCC.

## Methods

### Literature search strategy

A systematic literature search of the PubMed database was performed to identify all randomized controlled trials (RCT) of sorafenib or SBRT for unresectable hepatocellular carcinoma from January 1, 1999, to March 31, 2016. The search strategy was based on combinations of (“unresectable” or “inoperable” or “advanced” or “metastatic” hepatocellular carcinoma” [Mesh]) and (SBRT or sorafenib [Mesh] (“randomized controlled trials” or” clinical trials” [Mesh]). We also searched cost-effectiveness studies using the medical subject headings or text key words: quality-adjusted, QALY, life-year gained (LYG) and cost-effectiveness. The appropriate full text was reviewed and evaluated by 2 reviewers (AC and HL) independently, using the same inclusion and exclusion criteria. RCT or clinical studies published in English that evaluated sorafenib or SBRT for mHCC were included. Letters to the editor, case reports, non randomized trials, animal studies, editorials and posters were excluded. Any discrepancies in inclusion were resolved by consensus. We finally selected one RCT and one clinical trial of sorafenib and SBRT for advanced hepatocellular carcinoma as the clinical data source of the model.

### Markov model

**Fig. 1:**
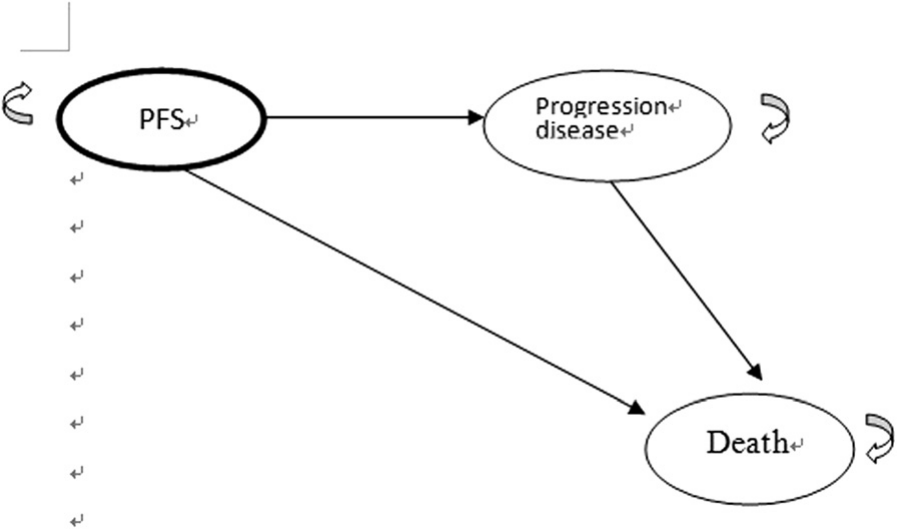
The decision-analytic, Markov model schema. PFS, progression free survival

The decision-analytic Markov model comparing the cost-effectiveness of two different regimens over a 5-year time horizon was programmed in TreeAge Pro 2014 Suite (R1.0 Released; TreeAge Inc., Williamstown, MA). The clinical data of population modeled, adverse events and treatment protocol were based on the SHARP and Phase I/II clinical trials [12, 16]. As the experts’ opinion and referred to the published literature, three health states were considered in the model: stable disease, progression disease and death for inoperable advanced HCC (Fig. 1). A patient in the model was considered to be in one of three health states at any time. All patients began from the stable stage and transit from one state to another on the basis of the transition probabilities and received either sorafenib or SBRT according to the treatment regimen. All parameters were detailed in Table 1. Our model did not include deaths from natural causes occurring in any health state. Death from cancer was assumed to happen after disease progression. The model perspective was based on that of the National Health Insurance (NHI) in Taiwan, with a 1-month cycle length and adjusted to half-cycle in each health state process. The model time horizon was set to 5 years to avoid the exclusion of long-term survivors. All costs and health outcomes were discounted at a real annual rate of 3 % to adjust for the relative value of the Taiwan dollar at present. We used the definition of willingness to pay (WTP) threshold suggested by the World Health Organization (WHO): 3 times the per capita gross domestic product (GDP) [19, 20]. The Taiwan per capita GDP in 2015 was $ NT$737,715 (US$22,355) [21]; thus, a WTP threshold was considered as $ NT2,213,145/QALY. The outcomes of the analysis are expressed as incremental costs effectiveness ratio (ICER) which was the costs spent to gain a quality adjusted life-years (QALY). Model parameters were described in Table 1. A panel of local experts (blood oncologist and radiation oncologist and one expert in economic evaluations) was consulted to ensure that assumptions taken into consideration in the model reflected routine clinical practice.

**Table 1:** Base-case values and ranges used in sensitivity analyses (± 30 %)

Parameters	Base estimate	Lower Limit- Upper Limit	Assumed distribution
Transition Probability			
PFS for sorafenib	0.1533	0.1073–0.1993	Beta
PFS To PD for sorafenib	0.0627	0.044–0.082	Beta
PD To death for sorafenib	0.1184	0.063–0.154	Beta
PFS for SBRT	0.086	0.061–0.034	Beta
PFS To PD for SBRT	0.109	0.076–0.142	Beta
PD To death for SBRT	0.0399	0.028–0.052	Beta
Utility			
PFS for sorafenib	0.76	0.546–1.014	Beta
PD for sorafenib	0.68	0.476–0.806	Beta
PFS for SBRT	0.72	0.504–0.936	Beta
PD for SBRT	0.63	0.441–0.819	Beta
Direct Medical Costs (US$ = 33 NT)			
cPFS for sorafenib	563365	394355–732375	Constant
cPFS for SBRT	259056	181339–336773	Constant
cPD for sorafenib	417528	292270–542786	Constant
cPD for SBRT	417528	292270–542786	Constant

*Abbreviations*: *c* cost, *SBRT* sterotactic body radiation therapy, *PFS* progression free survival, *PD* progression disease

### Treatment regimen

The outcome data from the SHARP and Phase I/II clinical trial were used [12, 16]. As expert opinions, the total radiation dose, sorafenib dose and schedule, patterns of treatment failure and survival of the regimen were assumed to be the same as those in the trials. According to the SHARP trial, patients were randomized to receive continuous sorafenib oral treatment with either 400 mg of sorafenib (consisting of two 200-mg tablets) twice daily for 6 week cycles. Tumor measurements were performed at screening, every 6 weeks during treatment (within 10 days before the end of each cycle), and at the end of treatment by computed tomography or magnetic resonance imaging. Patients visited the clinic every 3 weeks and at the end of treatment for assessment of compliance, safety, and determination of side effects. The SBRT treatment regimen was according to the phase I/II trial, doses of 30 to 54 Gy (24 to 54 Gy in Trial 1) in six fractions every other day over 2 weeks were delivered to the planning target volume (PTV). The dose to tumor vascular thrombosis plus PTV margin could be limited to 30 Gy. Detailed description was in the trial [16].

### Probabilities and utilities

Key probabilities for this model were included in Table 1. The trial did not collect probabilities and utility weights, therefore, the utilities were necessary to retrieve from the published literature [17, 18]. Transition probabilities of health states were estimated as follows: *P*(1 month) = 1- (0.5) ^(1/median PFS)^; this equation was derived from the following equations: *P* = 1 − e^− R^and*R* = − ln 〔0.5 〕/1 − (0.5)^(1/median time)/number of treatment cycles)^ [22, 23]. The efficacies of sorafenib and SBRT treatment were extracted by Kaplan-Meier survival curves of the patients whose data were collected from two clinical trials.

### Medical costs

The direct medical costs in this study were extracted from the National Health Insurance research database (NHIRD) and converted to 2015 NT dollar (1US$ = NT$ 33.0).The direct medical cost in this study included drug costs, laboratory test, physician visits, pharmacy dispensing fee, administration and nursing care fee. The drug costs for sorafenib and the costs of treatments for grade 3/4 adverse events (AE) were also included (Table 2). As a policy issued by the NHI, the reimbursement cost of SBRT is about NT$210,000 as a treatment package for early hepatocellular carcinoma and lung cancer, we therefore assumed this reimbursement cost for advanced HCC. We also assumed that the patient who survived by additional 1 month may produce the additional costs of visiting outpatient monthly.

**Table 2:** Estimated cost inputs used in the model

Cost input	Value	
Cost administration and health States		
PFS per course		
	Sorafenib	SBRT
Drug or treatment cost	537,264	210,000
Costs of test: laboratory test, CT	21,000	32,347
Costs of physician visit, dispensing fee, nursing care	3000	0
Adverse events treatment	38	16709
Sub-total	563,365	259,056
PD per visit		
Costs of test: laboratory test, CT	10,728	10,728
Costs of physician visit, dispensing fee, nursing care	15,600	15,600
End of life care	391,200	391,200
Sub-total	417,528	417,528

*Abbreviation*: *PFS* progression free survival, *PD* progression disease, *CT* computerized tomography, *SBRT* stereotactic body radiotherapy

### Sensitivity analysis

One-way sensitivity analysis was conducted to determine the potential impact of different parameters on this analysis; the result was presented as a tornado diagram. We hypothesized that the parameters varied over a range of ± 30 % in relation to its base-case value (Table 1).

The probabilistic sensitivity analysis using a Monte Carlo simulation was conducted to assess the impact of the uncertainty around the key parameters of the model on the ICER. That is, distributions for each parameter with the probabilistic sensitivity analysis were modeled. Log-normal distributions were adopted for all costs and beta distributions were adopted for probabilities, utilities and toxicity. The probabilistic sensitivity analysis was based on 10,000 samples, and the results were presented as a cost-effectiveness acceptability curve.

### Toxicity

We considered only grade 3–4 toxicity associated with sorafenib or SBRT as the two clinical trials. The rate of drug-related severe toxicity caused permanent treatment and discontinuation of the drug was 11 % for sorafenib and 24.8 % for SBRT [12, 16].

## Results

### Patient baseline characteristics

The baseline characteristics of the patients treated with sorafenib or SBRT were well balanced and no significant differences were noted with respect to sex, age, ECOG performance status, plasma levels of α-fetoprotein (AFP), Barcelona Clinic Liver Cancer staging B and C. However, the percentage of Child-Pugh class A liver function, extrahepatic spread and vascular invasion were higher in SHARP trial compared to the Phase I/II trial (Table 3).

**Table 3:** Baseline characteristics of the patients in the sharp and phase I/II trial

Characteristics	SHARP trial	Phase I/II trial	*P* value
Age, years	64.9	69.4	0.22
Male no (%)	260 (87 %)	80 (78.4 %)	0.244
Underlying liver disease			0.011
Hepatitis B	56 (19 %)	39 (38.2 %)	
Hepatitis C	87 (29)	39 (38.2 %)	
Alcohol related	79 (26 %)	25 (24.5 %)	
Other	28 (9 %)	14 (13.7 %)	
Unkown	49 (16 %)	7 (6.9)	
ECOG performance status n (%)			0.225
0	161 (54 %)	85 (83.3)	
1	114 (38 %)		
2	24 (8 %)	11 (10.8 %)	
BCLC stage			0.208
B (intermediate)	54 (18 %)	35 (34.3 %)	
C (advanced)	244 (82 %)	67 (65.7 %)	
Child-Pugh class, no (%)			< 0.0001
A	284 (95 %)	102 (100 %)	
B	14 (5 %)	0 %	
Biochemical analysis			0.25
Albumin (g/dl)	3.9	4.0	
Total bilirubin (mg/dl)	0.7	1.3	
Alpha-fetoprotein	44.3 ng/ml	163 nmol/L	
Previous therapy			0.079
Surgery	57 (19 %)	9 (8.8 %)	
TACE	86 (29 %)	22 (21.6 %)	
RFA	17 (6 %)	35 (34.3 %)	
PEI	28 (9 %)	16 (15.7 %)	
Extrahepatic spread (no,%)	159 (53 %)	12 (11.8 %)	< 0.0001
Vascular invasion (no,%)	108 (36 %)	20 (49 %)	< 0.0001

*Abbreviations*: *ECOG* Eastern Cooperative Oncology Group, *BCLC* Barcelona Clinic Liver Cancer staging system, *TACE* transarterial chemoembolization, *RFA* radiofrequency ablation, *PEI* percutaneous ethanol injection

### Direct medical costs

The direct medical costs of the drugs, laboratory test, physician visits, pharmacy dispensing fee, administration, nursing care fee and grade 3/4 AE treatments were shown in Table 2.

### Effectiveness

**Fig. 2:**
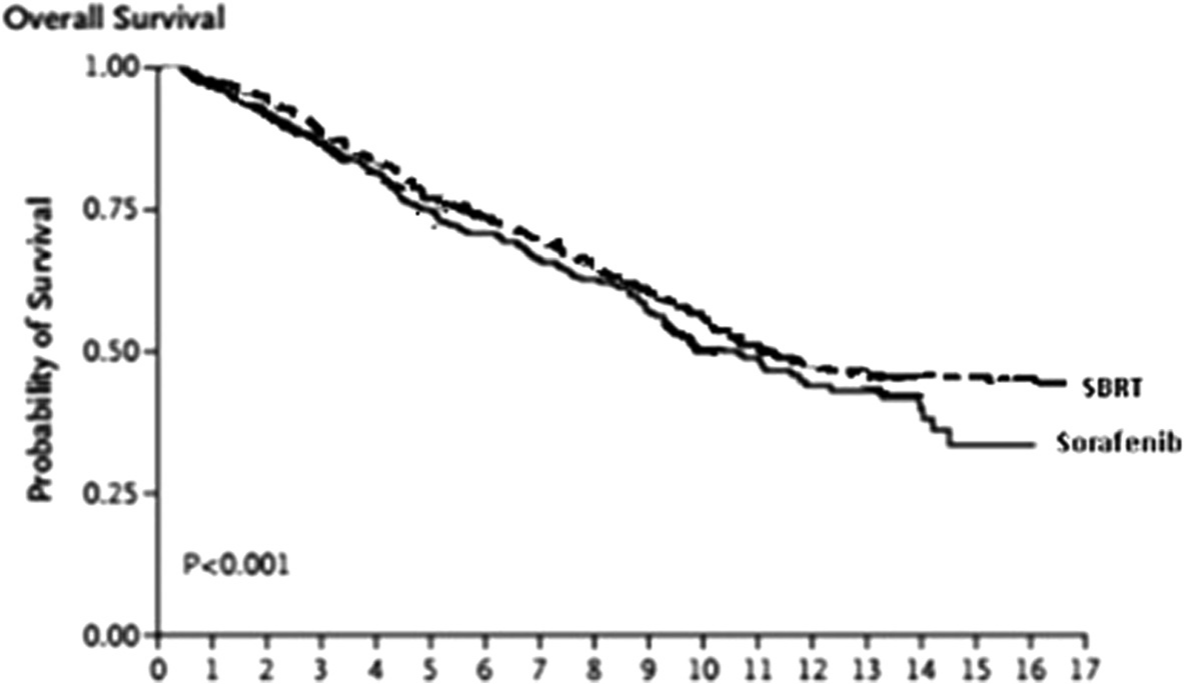
Kaplan-Meier Analysis of Overall survival

The median overall survival (mOS) and median time to progression (mTTP) were 10.7 and 4.1 months in the sorafenib group of the SHARP trial, whereas, the mOS and mTPP were 17 and 6.0 months in the SBRT group from the Phase I/II trials, as shown in the Kaplan-Meier survival curves (Fig. 2). According to the equation described above, the monthly transition probability for PFS state was 0.153, from PFS to progression disease (PD) state was 0.0627, from PD state to death was 0.1184 for the sorafenib group. For SBRT, the monthly transition probability for PFS state was 0.086, from PFS to progression disease (PD) state was 0.109, from PD state to death was 0.0399 (Table 1). Utility scores of the health states were retrieved from two previously published studies (sorafenib group: 0.68 for PD, 0.76 for PFS; SBRT group: 0.63 for PD_1_, 0.72 for PFS_1_) [24, 25].

### Base-case analysis

The cost-effectiveness analysis demonstrated that the ICER for sorafenib compared to SBRT was NT$ 3,788,238 per quality-adjusted life year gained (cost/QALY) in the base scenario. Based on the WTP threshold of NT$ 2,213,145/QALY, the sorafenib was not cost-effective versus SBRT under the defined WTP threshold in this analysis (Table 4).

**Table 4:** Incremental Cost-Effectiveness Ratios Comparing SBRT versus sorafenib at the Base Case

Various cost	Sorafenib	SBRT
QALYs (years)	3.07	2.81
Incremental QALY gained (years)	0.26	-
Lifetime cost (US$)	2166,079.7	1,197,039.2
Incremental cost (NT$)	969,041	-
Cost/effectiveness	704,857.96	426,117.13
ICER (NT$)	3,788,238	
Cost-effectiveness threshold (NT$)	2,213,145	
Is SBRT cost-effective?	No	

### Sensitivity analyses

**Fig. 3:**
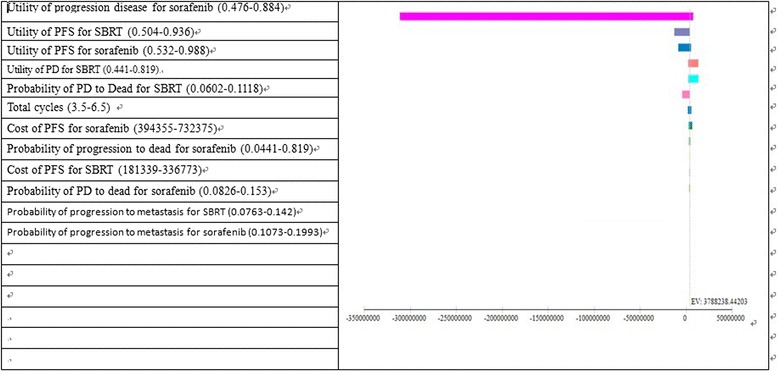
Tornado analysis (ICER) for SBRT vs sorafenib. EV, expect value of ICER for gem + IMRT

One-way sensitivity analysis was done for SBRT and sorafenib with a hypothesized variation of ±30 % in patients treated with two regimens by using the tornado diagram. The results showed the important parameters driving the model. The broader the horizontal bar in a tornado diagram, the more impact the input parameter has on the model. Analyses showed that the results of the model were highly sensitive to an assumption on the first five parameters, which were utility of progression disease for the sorafenib treatment, utility of progression free survival for SBRT, utility of progression free survival for sorafenib, utility of PFS to progression disease for SBRT and transition probability of progression disease to dead for SBRT (Fig. 3).

### Probabilistic sensitivity analyze

**Fig. 4:**
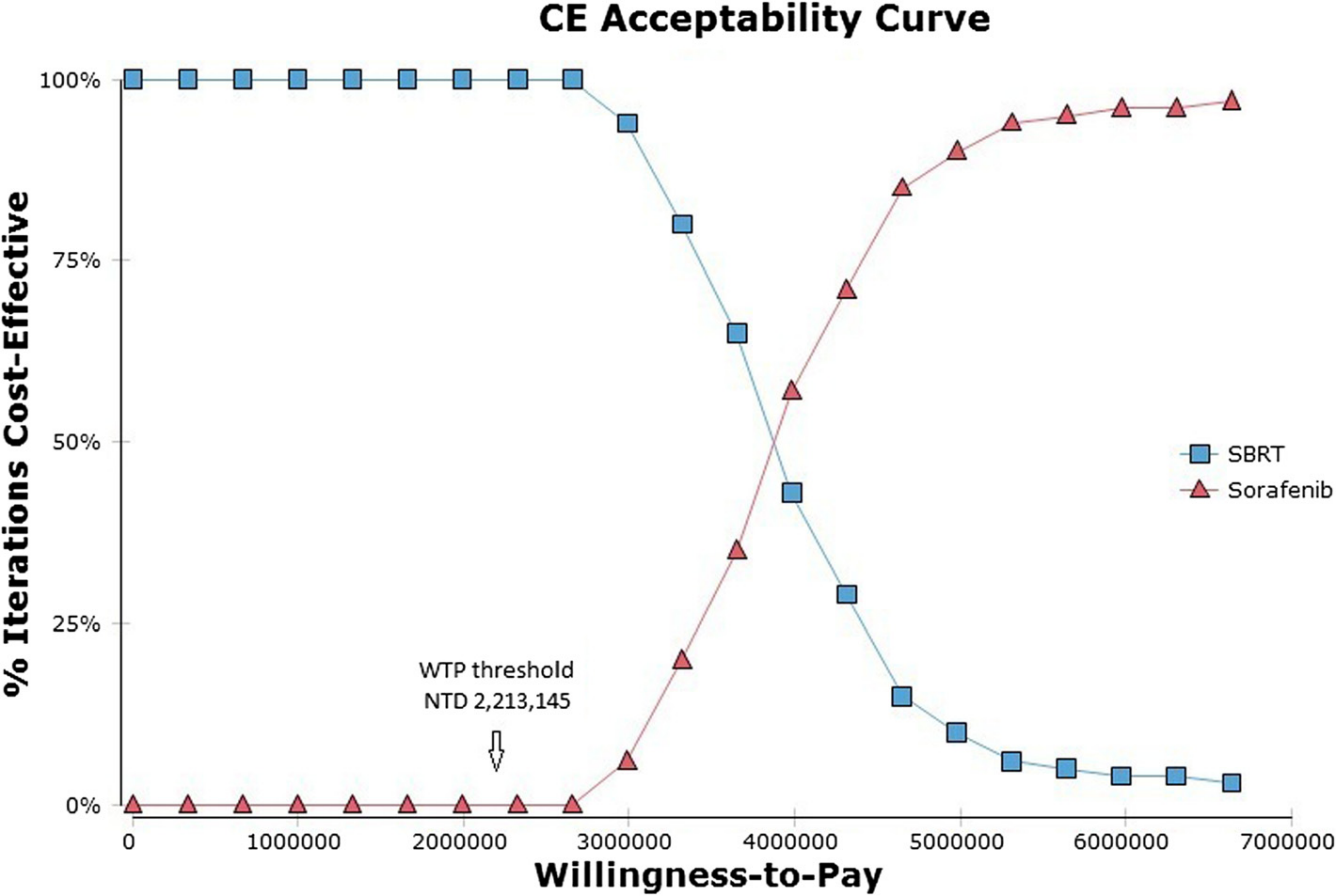
Cost-effectiveness acceptability curve. These probability that a specific treatment is cost-effective at a given Willingness-to-pay threshold of NT 2,213,145 (=US$ 67065, 1 US = 33 NT). SBRT, stereotactic body radiotherapy

Varying all variables simultaneously by ± 30 % in the Monte Carlo simulation, the results demonstrated that the probability of cost-effectiveness at a willingness to pay threshold of NT$ 2,213,145 per QALY was 100 % and 0 % chance for SBRT and sorafenib (Fig. 4).

## Discussion

Sorafenib is currently used as the most effective option for patients with advanced un-resectable HCC and for those contraindicated to TACE [12]. The median overall survival of patients treated with sorafenib was 10.7 months. Most of the economic evaluation on sorafenib in unresectable HCC was cost-effective compared to best supportive care conducted in the USA [26, 27]. However, it was not a cost-effective option for patients with advanced HCC from the societal perspective in China [25]. Due to advances in radiotherapy planning and imaging technologies, SBRT have been suggested to be used safely for localized or unresectable advanced HCC with local control rate of 75–100 % at 1 to 2 years. Additionally, SBRT may provide better quality of life because of more favorable toxicity profile [16]. As the single payer Healthcare system in Taiwan, the high drug cost of sorfenib used for treating patients with advanced HCC have become one of the biggest issues. The decision-maker is probably willing to pay for less expensive and high outcome treatment for these patients. The result of this study is probably to provide the healthcare payer with evidence in determining a reasonable reimbursement price for the effective treatment strategy for patients with advanced HCC.

In our study, patients in the sorafenib group gained 3.07 QALYs at a cost of NT$ 2,166,079; patients in the SBRT group gained 2.81 QALYs at a cost of NT$1,197,039. Sorafenib increased 0.26 QALYs for these patients but with an incremental cost of NT$969,041 compared to SBRT. The ICER in our study was NT$ 3,788,238/QALY(US$114,795/QALY) for sorafenib versus SBRT treatment, which was significantly higher than the defined societal willingness to pay threshold in our country (NT$2,213,145/QALY = US$67,065/QALY) (Table 4). Therefore, sorafenib is not a cost-effective treatment for patients with unresectable advanced HCC in Taiwan. This result is consistent with the Zhang et al. study conducted in China because the dose of sorafenib used in their study was similar to that used by our oncologists in the field practice [25]. Furthermore, they also used the same definition of WTP as WHO’s guideline. However, the ICER in the Zhang et al. study was higher than that in our study. The reason may be the high percentage of vascular invasion in the patients collected in their study (macroscopic vascular invasion was 47.9 % versus ours 36 %, no data available to compare extrahepatic spread), which likely lead to a more unfavorable outcome in the sorafenib group in terms of TTP, OS and utility.

The high drug cost of sorafenib may be one of the key variables that have a great impact on the ICER as compared to placebo or other alternative regimen. The price of sorafenib reimbursed by the National Health Bureau was calculated based on the market price and negotiated with the manufacturer. The difference in the drug cost and other direct medical cost may result in different ICER value. On the contrary, SBRT is a cost-effective option for patients with unresectable advanced HCC under the assumption of reimbursement in our study. One-way sensitivity analysis revealed SBRT to be cost-effective regardless of the variation in any single parameter. As our expert opinion, the fixed reimbursement price of NT$ 210,000 per case may not affect the dose or technique of SBRT used for either early or unresectable advanced HCC. Therefore, from the perspective of single payer healthcare system, SBRT for the treatment of unresectable advanced HCC is likely to be cost-effective compared to sorafenib,

This study has some limitations. First, the perspective of our study was not societal. Only direct medical costs were involved, therefore, the costs may be underestimated. Second, our study did not consider dose adjustments (interruptions or reductions), it may have an impact on ICER value in sorafenib group. Third, the percentage of extrahepatic spread in patients of SHARP trial was higher than that in Phase I/II SBRT trial. This difference in patient characteristic is likely lead to a more unfavorable outcome in the sorafenib group in terms of TTP and OS, thus may have negative impact on the utility value. Fourth, the percentage of Child-Pugh class A liver function of patients recruited in SHARP trial and Phase I/II SBRT trial were 95 and 100 %. As reported by the GIDEON study [28], patients with advanced HCC with Child-Pugh class A liver function had a longer OS and TPP than those with Child-Pugh class B liver function. This difference may slightly have influence on the utility. However, the uncertainties associated with these limitations have been estimated by varying wide ranges of input parameters in sensitivity analyses and the results are robust.

## Conclusion

Patients with unresectable advanced HCC within the patients’ criteria in our study, SBRT is more cost-effective than sorafenib in Taiwan.

### Ethics approval and consent to participate

This work did not require any written patient consent. The local ethics committee of Centre Jean Perrin approved this work.

### Consent for publication

Not applicable.

### Availability of data and materials

The datasets supporting the conclusions of this article are included within the article.

## Abbreviations

BCLC: Barcelona Clinic Liver Cancer staging system
EGOG: Eastern Cooperative Oncology Group
ICER: incremental cost effectiveness ratio
mHCC: metastasis hepatocellular carcinoma
mOS: median overall survival
mTTP: median time to progression
*P*: transition probability
PD: progression state
PFS: progression free survival
PTV: planning target volume
QALY: quality adjusted life year
RCT: randomized controlled trial
SBRT: stereotactic body radiotherapy
WTP: willingness to pay
